# The moderating role of stigma in the relationship between depression and resilience: results of a cross-sectional study in university students

**DOI:** 10.3389/fpsyg.2024.1392381

**Published:** 2024-04-15

**Authors:** Caroline Rometsch, Giovanni Mansueto, Sara Ceccatelli, Fiammetta Cosci

**Affiliations:** ^1^Department of Experimental and Clinical Medicine, University of Florence, Florence, Italy; ^2^Department of Health Sciences, University of Florence, Florence, Italy; ^3^School of Applied Sciences, London South Bank University, London, United Kingdom; ^4^Department of Psychiatry and Neuropsychology, Maastricht University, Maastricht, Netherlands

**Keywords:** mental health, stigmatization, university, resilience, students

## Abstract

**Background/objective:**

Depression is a growing concern in university students and resilience has shown to play a protective role. The impact of stigma is still under-explored, with reference to its moderating role between depression and resilience. The present study investigate such a relationship among Italian university students.

**Methods:**

A cross-sectional design was applied in a simple of 1,912 students to examine the interrelationships between depression (Patient Health Questionnaire-9), resilience (Nicholson McBride Resilience questionnaire), and stigma (Stigma-9). Correlation, predictor, and moderation analyses were applied in RStudio.

**Results:**

A negative correlation was found between depressive symptoms and resilience (*r* = −0.455, *p* < 0.001). A positive correlation was found between depressive symptoms and stigma (*r* = 0.207, *p* < 0.001). Lower levels of resilience and higher levels of stigma were significant predictors of depressive symptoms [*F*_(df, n)_ = 190.8_(3, 1884)_, *p* < 0.001, *R*^2^ = 0.236]. The moderation analysis showed a weakening of resilience protective effect against depression as stigma levels increase [*F*_(df,n)_ = 186.7_(3,1908)_, *p* < 0.001, *R*^2^ = 0.226].

**Conclusion:**

Stigma influences the relationship between depression and resilience. Anti-stigma interventions and programs empowering resilience, should be implemented in university settings to protect students from depression.

## Introduction

Depression is a significant concern among university students with a point prevalence of about 27% (Rotenstein et al., 2016). It is linked to the demands of studies (Frajerman et al., 2019) and correlates with poorer academic performance and health-threated behaviors (Sharp and Theiler, 2018). Resilience, the ability to maintain the persistence of one’s orientation toward existential purposes (Sisto et al., 2019), has emerged as an essential protective factor against depression, being the individual dynamic ability to adapt successfully to adverse situations (Link and Phelan, 2001). Students with low resilience notably experience higher psychological distress (Bacchi and Licinio, 2017), which in turn decreases well-being (Li and Hasson, 2020), and more severe depressive manifestations (Ahmed and Julius, 2015; Kelifa et al., 2021).

The interplay between depression and resilience is rather complex and involves different variables, including stigma (Crowe et al., 2016). Stigma has to do with shame, penalty, or dishonor (Economou et al., 2020). An individual is stigmatized when he/she is deeply discredited and rejected by the society due to a specific attribute (Goffman, 2009). A person can be stigmatized for his/her mental or physical attributes/signs, which are perceived as socially undesirable or unacceptable. The presence of stigma can result in individuals experiencing exclusion, discrimination, and marginalization (Goffman, 2009). Stigma has a multifaced nature (Link and Phelan, 2001) referring to: (1) labeled differences, that is distinguishing and categorizing human variations; (2) stereotypes, which are associated with negative generalizations; (3) separation, which defines the divide between the mainstream and the marginalized; (4) status loss and discrimination, emphasizing the disadvantages faced by those marginalized; (5) power, underscoring the societal hierarchies that perpetuate discrimination; and (6) emotional reactions, capturing feelings from shame in the stigmatized to disgust in the stigmatizers (Link and Phelan, 2001). Stigma is a widespread phenomenon among university students, with prevalence rates ranging from 21 to 97% (Tzouvara et al., 2016; Zolezzi et al., 2017; Gervas et al., 2020). Stigma influences individuals’ perception of responsibility regarding the emergence and maintenance of their health status and may impact resilience (Boardman et al., 2011). Higher levels of stigma might motivate students to develop greater resilience (Boardman et al., 2011) but may also inhibit help-seeking behaviors among those with depression (Suwalska et al., 2017).

While the conceptual evolution of stigma and its implications for mental health have been extensively explored, the interplay between depression and resilience in university students, with stigma as a moderating variable, has never been examined. The present study applied a cross-sectional design to investigate such a relationship among Italian university students. We hypothesize: (a) at least a moderate correlation between depression, resilience, and stigma; (b) a direct moderating role of stigma in the relationship between depression and resilience.

## Materials and methods

### Study design

A cross-sectional online survey was conducted at the University of Florence, Italy. On May 13, 2022, an invitation with an embedded link to the online survey was spread at the institutional email addresses of the students and remained accessible until May 30, 2022. All participants received instructions (i.e., a brief overview of the research, involved investigators, survey content and duration, and assurance of anonymity) and submitted their informed consent through an online form. The study, entitled Mental Health Literacy among students (MATTERS), was granted by the European University for Well-being (EUniWell) Consortium, as part of the 2021 Seed Funding’s second call. The study was approved by the local Ethical Committee (document n. 184, November 23, 2021). All procedures contributing to this work comply with the ethical standards of the relevant national and institutional committees on human experimentation and with the Helsinki Declaration of 1975, as revised in 2013.

### Sample description

Students aged 18 and above enrolled at the University of Florence in the 2021–2022 academic year with a working institutional email were qualified to participate. No exclusion criteria were applied. Involvement was on a voluntary basis and participants received no compensation.

### Assessment instruments

The Patient Health Questionnaire (PHQ-9) (Kroenke et al., 2001) is a self-administered questionnaire of the PRIME-MD tool. It focuses on depression and evaluates each of the nine DSM-IV criteria for major depression on a four-point Likert scale from “0” (not at all) to “3” (nearly every day). PHQ-9 total score ranges from 0 to 27 to categorize depression severity. Scores from 0 to 4 indicate minimal depression, those between 5 and 9 suggest mild depression, individuals obtaining scores from 10 to 14 are classified as having moderate depression, scores from 15 to 19 represent moderately severe depression, whereas scores that fall between 20 and 27 imply severe depression. The PHQ-9 has shown satisfactory clinimetric properties (Cosci et al., in press).

The Nicholson McBride Resilience questionnaire (NMRQ) (McBride, 2020) is a 12-item, self-administered tool designed to quantitatively assess resilience using a five-point Likert scale ranging from “1” (strongly disagree) to “5” (strongly agree). The sum score is obtained by adding the individual scores of the 12 items, providing a comprehensive measure of resilience. A total score ranging from 0 to 37 identifies a developing (i.e., evolving) resilience. A total score of 38–43 indicates an established resilience, with occasional challenges. A total score of 44–48 represents a strong resilience, with a capacity for adaptive recovery. A total score of 49–60 means an exceptional resilience, indicating consistent robustness against adversities. The tool showed high validity (Pilafas et al., 2020).

The Stigma-9 (STIG-9) (Gierk et al., 2018) is a self-report measure developed to assess mental health-related stigma. STIG-9 showed a single-factor structure, supporting the utilization of a total score (range 0–27) for data synthesis. Higher STIG-9 scores positively correlated with decreased mental quality of life, enhanced social impairment, and more severe depression. No association was found between somatic symptom burden and STIG-9 scores (McBride, 2020).

### Statistical analysis

Sociodemographic data and validated instruments scores were analyzed descriptively. To determine the randomness of missing data, the Little’s missing completely at random (MCAR) test was conducted using the package *naniar*. Findings showed data are missing completely at random for PHQ-9 [χ^2^_(df)_ = 28.7_(26)_, *p* = 0.327], NMRQ [χ^2^_(df)_ = 7.83_(11)_, *p* = 0.728], and STIG-9 [χ^2^_(df)_ = 49.7_(38)_, *p* = 0.097]. Given this diversity in missing data patterns, the robust imputation method multiple imputations by chained equations (MICE) was used, leveraging the *mice* package. The Predictive Mean Matching (PMM) technique was applied. For each scale, five imputed datasets were generated using 50 iterations to ensure convergence. The product–moment correlation was conducted between PHQ-9, NMRQ, and STIG-9 total scores. A multiple linear regression analysis was applied to discern the relationship between the dependent variable (i.e., PHQ-9) and the independent variables (i.e., NMRQ, STIG-9) using the *stats* packages and the *corr.test()* and *lm()* functions. Statistical assumptions for using multiple linear regression analyses were evaluated. Multicollinearity was deemed not to be problematic for the data set since the tolerance index and variable inflation factors ranged from 0.973 to 1.027 (Sen and Srivastava, 1997; Hinkle et al., 2003). An analysis was conducted to analyze whether stigma (STIG-9) moderates the relationship between resilience (NMRQ) and depression (PHQ-9) using the *lm()* function. To test moderation model, three regression equations were run and the following conditions verified: (a) the independent variable (i.e., NMRQ) affects the dependent variable (i.e., PHQ-9); (b) the moderator variable (i.e., STIG-9) affects the dependent variable (i.e., PHQ-9); (c) the interaction effect between independent (i.e., NMRQ) and moderator (i.e., STIG-9) variables affects the dependent variable (i.e., PHQ-9) (Baron and Kenny, 1986).

Statistical analyses were run in RStudio (version 4.3.0).

## Results

### Sociodemographic and clinical data

A total of 1,912 subjects participated in the survey and completed PHQ-9, NMRQ, and STIG-9. The average age was 24.02 years (*SD* = 3.83). Most of them (*N* = 1,061; 55.49%) were pursuing an undergraduate degree. Counseling services were sought by 645 (33.7%) students, with 422 (22.1%) using psychological counseling. Of them 314 (16.4%) engaged in multiple counseling sessions. About half of the participants (*N* = 977; 51%) contemplated/initiated psychotherapy, 444 (23.2%) were actively undergoing psychotherapy, and 375 (19.6%) were involved in psychiatric treatment (see Table 1 for details).

**Table 1:** Sociodemographic and clinical variables (*N* = 1,912).

	*N*	%
Sociodemographic variables		
Nationality		
Italian	1,843	96.4
Other than Italian	69	3.6
Study information		
Full-time student	1,624	84.9
Part-time student	169	8.8
Week-end workers	119	6.2
Financial support		
Family	1,474	77.1
Job	555	29.0
Fellowship	213	11.1
Credit	18	0.9
Living situation		
With parents	1,238	64.6
With a friend	344	18.0
With spouse	201	10.5
Alone	87	4.6
Shard housing	40	2.0
Children		
No	1,850	96.8
Yes	62	3.2
Clinical variables		
Mental illness in family		
Yes	701	36.7
No	698	36.5
Not sure	512	26.7
Mental illness in friends		
Yes	905	47.4
No	499	26.1
Not sure	507	26.5

**Figure 1:**
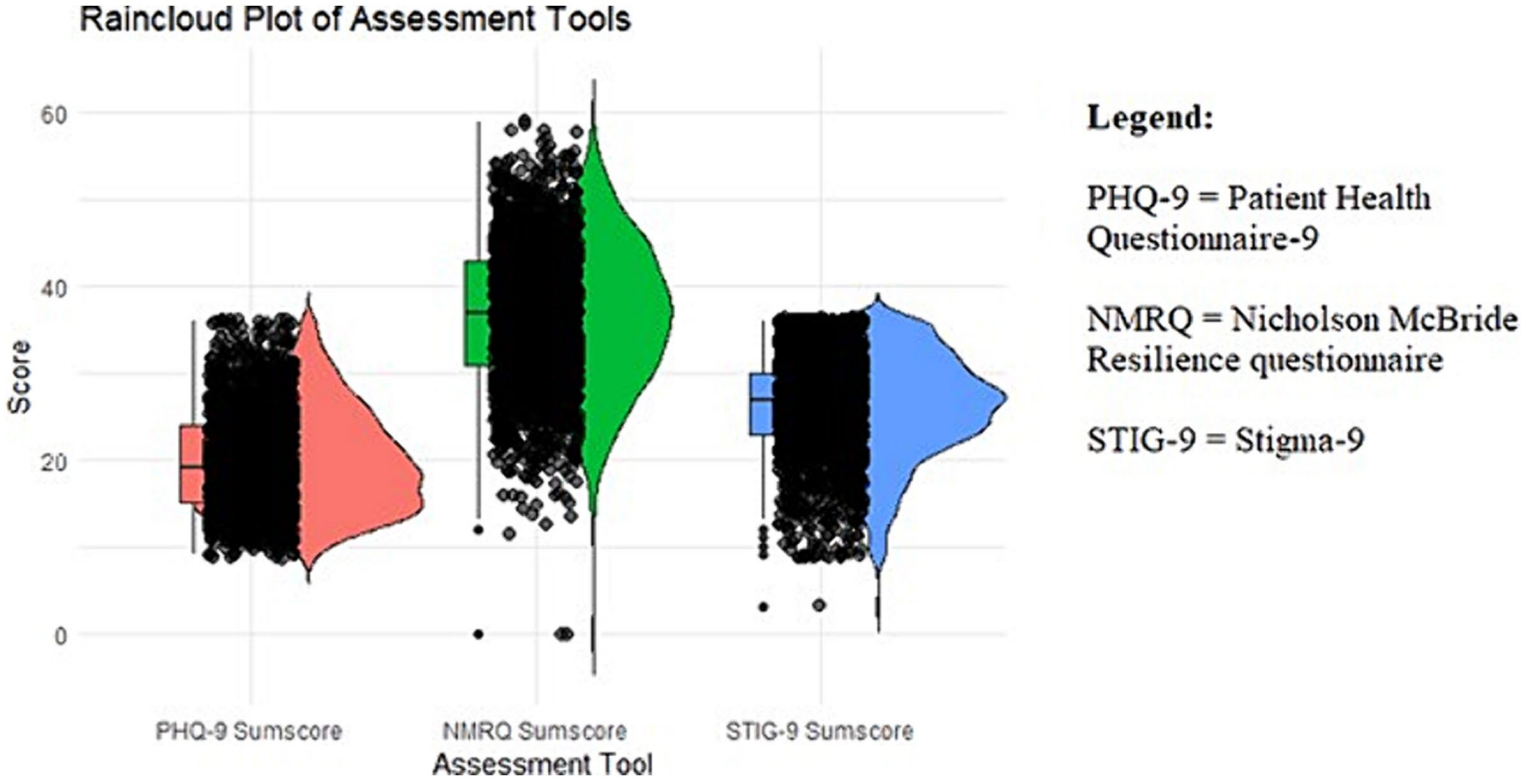
Graphical representation of Patient Health Questionnaire (PHQ-9), Nicholson McBride Resilience questionnaire (NMRQ), and Stigma-9 (STIG-9) total mean scores: PHQ-9 total mean score was 19.84 (SD = 5.79); NMRQ total mean score was 36.62 (SD = 8.27); and STIG-9 total mean score was 26.23 (SD = 5.85).

Patient Health Questionnaire total mean score was 19.84 (*SD* = 5.79). Twelve (0.6%) students presented mild depression, 370 (19.4%) moderate, 637 (33.3%) moderately severe depression, and 893 (46.7%) severe depression. NMRQ total mean score was 36.62 (*SD* = 8.27). Most of the participants (1,025, 53.6%) had an evolving level of resilience, followed by 477 (25%) who exhibited an established level of resilience, while 270 (14.1%) displayed a strong level of resilience, and 140 (7.3%) an exceptional level of resilience. STIG-9 total mean score was 26.23 (*SD* = 5.85) (see Figure 1 for graphical representation of PHQ-9, NMRQ, and STIG-9 total scores).

### Correlation analysis

The relationship between PHQ-9 and NMRQ total scores was found to be negative and moderately robust (*n* = 1,910; *r* = −0.455, *p* < 0.001, 95%CI: [−0.490 to −0.419]) as well as between STIG-9 and NMRQ (*n* = 1,910; *r* = −0.163, *p* < 0.001, 95%CI: [−0.206 to −0.119]). A positive and weak correlation was found between PHQ-9 and STIG-9 total scores (*n* = 1,910, *r* = 0.207, *p* < 0.001, 95%CI: [0.164–0.250]).

### Moderation analysis

The regression analysis aimed at understanding the influence of resilience and stigma on depression was statistically significant with *F*_(n, df)_ = 277.6_(2, 1909)_, *p* < 0.001. The stronger the resilience (NMRQ), the fewer the depressive symptoms (PHQ-9) were observed. An increased stigma score (STIG-9) was associated with more severe depressive symptoms (PHQ-9). The regression model resulted in an adjusted *R*-squared value of 0.2245, implying that approximately 22.45% of the variance of PHQ-9 was explained by NMRQ and STIG-9 scores [*F*_(n, df)_ = 277.6_(2, 1909)_, *p* < 0.001] (see Table 2).

**Table 2:** Independent predictors of depression (PHQ-9) among Italian university students.

Variable	β	SE	95% CI	*p*	VIF	TI
NMRQ	−0.303	0.014	−0.331 to −0.275	< 0.001^***^	1.027	0.973
STIG-9	0.135	0.020	0.095–0.174	< 0.001^***^	1.027	0.973

Multiple linear regression. β, Regression coefficient; SE, Standard deviation; CI, Confidence interval, VIF, Variable inflation factor; TI, Tolerance index. ^***^p < 0.001. NMRQ, Nicholson McBride Resilience Questionnaire; STIG-9, Stigma-9.

Based on these results, stigma (STIG-9) was tested as moderating factor in the relationship between depression (PHQ-9) and resilience (NMRQ). The linear regression model indicated a significant negative relationship between depression and resilience [β = −0.178, *t*_(n)_ = −2.770_(1908)_, *p* < 0.01]. Stigma was found to be positively associated with depression [β = 0.312, *t*_(n)_ = 3.416_(1908)_, *p* < 0.001]. The interaction term between resilience and stigma was significant [β = 0.005, *t*_(n)_ = −1.984_(1908)_, *p* < 0.05], which implies a moderating effect of stigma on the relationship between resilience and depression. The model accounted for approximately 22.67% of the variance of depression [*F*_(df,n)_ = 186.7_(3,1908)_, *p* < 0.001, *R*^2^ = 0.226] (see Table 3).

**Table 3:** Moderating effect of STIG-9 in the association between PHQ-9 and NMRQ among Italian university students.

	Variable	β	SE	*t value*	*p*	*95%CI*
Step 1	IV: NMRQ resilience	−0.178	0.064	−2.770	0.006^**^	−0.331 to −0.275
Step 2	MR: STIG-9 stigma	0.312	0.091	3.416	<0.001^***^	0.095–0.174
Step 3	Interaction term	0.005	0.002	−1.984	0.047^*^	

Moderation analyses according to Baron and Kenny criteria (1986). IV, Independent variable; MR, Moderator variable; β, Regression coefficient; SE, Standard deviation; CI, Confidence interval. ^*^p < 0.05, ^**^p < 0.01, and ^***^p < 0.001. NMRQ, Nicholson McBride Resilience Questionnaire; STIG-9, Stigma-9.

## Discussion

A cross-sectional study was conducted to analyze depression, resilience, and stigma among university students, and their interplay. A notable relationship was found between fewer depressive symptoms and higher resilience and between increased stigma and more severe depressive symptoms. Stigma played a moderating role in the relationship between resilience and depression, suggesting that as stigma increases, the protective effect of resilience on depression becomes weaker.

Depression has emerged as a prominent and pressing issue among university students over recent decades (Akhtar et al., 2020) and its relevance has increased also under the growing attention toward the so-called minority stress, that is the psychological distress experienced by minorities such as sexual and gender ones (Meyer, 2003). Identifying protective factors against depression has indeed become a priority (McBride, 2020). Consequently, concepts such as resilience have gained relevance to understand and aid students facing depressive symptoms (Liu et al., 2019), especially if they belong to stigmatized groups (e.g., immigrants, blacks, and gender-nonconforming individuals) (Messman and Leslie, 2019; Venta et al., 2019; Oshin et al., 2022). The present findings are consistent with the literature identifying low resilience as a predictor of depressive manifestations (Hjemdal et al., 2007).

When considering the role of stigma, our findings are consistent with the literature suggesting that higher levels of stigma may be associated with more severe depressive symptoms (Musa et al., 2020; Prizeman et al., 2023). A critical aspect appears to be the significant moderating role that stigma plays between depression and resilience. There is limited understanding of how stigma may impact the interplay between depression and resilience among university students. Findings only suggested that diminishing stigma could potentially enhance self-efficacy among them (Beasley et al., 2020).

Resilience is a recognized protective factor for depression, and numerous university programs have been designed to enhance it (Brewer et al., 2019; Walsh et al., 2020). However, based on the present results, the effectiveness of these programs should be evaluated under the light of the influence of stigma, which weakens the protective effect of resilience against depression. Stigma has also been associated with a negative mental help-seeking attitude among students, thus representing a barrier to get the right treatment (Ibrahim et al., 2019). In order to overcome such a barrier, universities should implement education and/or treatment programs against stigma (Clement et al., 2013; Lien et al., 2021), in addition to the anti-stigma campaigns (Giralt Palou et al., 2020; Pingani et al., 2021; Walsh and Foster, 2021), which showed weak or no significant long-term effects (Clement et al., 2013; Pingani et al., 2021; Walsh and Foster, 2021).

The present study has the strength to give the first overview on the role of stigma in the interplay between depression and resilience in university students. However, some limitations should be mentioned. The cross-sectional design restricts the possibility to offer insights into long-term trends; nonetheless, the large number of participants gives valuable initial insights and implications. The voluntary nature of participation may have selected those with higher stigma or depression and may have introduced self-report biases, however only via surveys is possible to collect such information in a large sample. Attention questions were not included in the survey form; however, missing data were random. Finally, while the study effectively highlights the moderating role of stigma, exploring additional potential mediators or moderators (e.g., social support, coping mechanisms) could offer a more comprehensive understanding of the factors influencing the depression-resilience nexus.

In conclusion, stigma is underappreciated in the challenge against depression among university students even though it weakens the strength of the relationship between depression and resilience. When stigma is not explicitly addressed, university students may not take full advantage of strategies aimed at targeting depression via an empowerment of resilience. Only through a combination of treatment and educational programs tailored to combat stigma (Giralt Palou et al., 2020; Pingani et al., 2021) and interventions addressing depression via resilience, university students with depressive symptoms might be effectively aided in their pursuit of a good health status.

## Data availability statement

The original contributions presented in the study are included in the article/supplementary material; further inquiries can be directed to the corresponding author.

## Ethics statement

The studies involving humans were approved by Commissione Etica di Ateneo, Università degli Studi di Firenze. The studies were conducted in accordance with the local legislation and institutional requirements. The participants provided their written informed consent to participate in this study.

## Author contributions

CR: Data curation, Writing – original draft. GM: Writing – review & editing. SC: Writing – review & editing, Data curation. FC: Data curation, Writing – review & editing, Conceptualization, Funding acquisition, Methodology, Project administration, Resources, Supervision, Validation.
